# Immune Responses to SARS-CoV2 Mirror Societal Responses to COVID-19: Identifying Factors Underlying a Successful Viral Response

**DOI:** 10.3390/biology10060485

**Published:** 2021-05-29

**Authors:** Shahar Lev-Ari, Benjamin Rolnik, Ilan Volovitz

**Affiliations:** 1 Tel-Aviv Sourasky Medical Center, Department of Health Promotion, School of Public Health, Sackler Faculty of Medicine, Tel-Aviv University, Tel-Aviv 69978, Israel; 2 Healthcare Innovation Lab, Department of Genetics, Stanford Medicine, Stanford, CA 94305, USA; rolnik@stanford.edu; 3 Tel-Aviv Sourasky Medical Center, Cancer Immunotherapy Lab, Neurosurgery Department, Tel-Aviv 64236, Israel

**Keywords:** adaptive immune system, COVID-19, social cytokine storm, biological analogy, cytokine storm, pandemic, public health, social immunity

## Abstract

**Simple Summary:**

The immune system was sculpted in numerous evolutionary battles to protect individuals, societies, and species from novel pathogens. During that time, it had developed highly effective strategies to cope with novel pathogenic challenges and retain immune “memory” following pathogen clearance. We found surprising parallels between person-level immune responses to the virus causing COVID-19 and the society-level responses to the COVID-19 pandemic. Similarly, the events which lead to a life-endangering immune cytokine storm, parallel those leading to a devastating socioeconomic crisis we termed a “social cytokine storm”. This new understanding may help explain why COVID-19, caused by a virus with no “exceptional” epidemiological capabilities, had inflicted such profound social and global toll. Understanding the set of events leading to an immunological and societal cytokine storms may aid scientists and policymakers to adopt more effective strategies that could reduce future disease morbidity and mortality, avert avoidable social cytokine storms, and minimize the socioeconomic toll.

**Abstract:**

The adaptive immune system was sculpted to protect individuals, societies, and species since its inception, developing effective strategies to cope with emerging pathogens. Here, we show that similar successful or failed dynamics govern personal and societal responses to a pathogen as SARS-CoV2. Understanding the self-similarity between the health-protective measures taken to protect the individual or the society, help identify critical factors underlying the effectiveness of societal response to a pathogenic challenge. These include (1) the quick employment of adaptive-like, pathogen-specific strategies to cope with the threat including the development of “memory-like responses”; (2) enabling productive coaction and interaction within the society by employing effective decision-making processes; and (3) the quick inhibition of positive feedback loops generated by hazardous or false information. Learning from adaptive anti-pathogen immune responses, policymakers and scientists could reduce the direct damages associated with COVID-19 and avert an avoidable “social cytokine storm” with its ensuing socioeconomic damage.

## 1. Immune Responses to SARS-CoV2 Mirror Societal Responses to COVID-19

COVID-19 is one of the most impactful pandemics in human history. From the first recorded case in November 2019 through May 2021 there have been more than 3.4 million confirmed COVID-19 deaths [1]. COVID-19 is the 3rd most lethal epidemic throughout the 20th and 21st centuries [2]. As for the proportion of global population lost, COVID-19 is, at most, at the 16th place throughout history with approximately 0.04% global mortality [1,3]. Notwithstanding, the entire world has been economically and socially impaired by it. How then, could a disease with a relatively low global mortality rate exact such a global toll?

The epidemiological parameters of COVID-19 cannot explain its profound social impact. Its basic reproduction number (R_0_), though debated, was calculated in a meta-analysis to be 2.87. This is within the range of H1N1 influenza, and considerably lower than that of measles or smallpox [3]. The fatality rate of SARS-CoV-2 (severe acute respiratory syndrome coronavirus 2) that drives COVID-19 varies between countries and demographics and considerably increases with age and specific comorbidities. Yet the overall fatality rate across all global COVID-19 confirmed cases is approximately 2.1% [1]. This fatality rate is considerably lower than that of related coronaviruses such as SARS-CoV-1 or the Middle East respiratory syndrome (MERS)-CoV. The mutability of SARS-CoV-2, possessing a proofreading enzyme, is slower than that of most RNA viruses and its speed of transmission compared to influenza (sharing transmission routes) is also slower [4,5]. Taken together, SARS-CoV-2 has no “exceptional” viral-related parameters that may explain its global toll. We propose here that an additional parameter may help explain the unique socioeconomic impact of COVID-19: its unique ability to drive a social-level “cytokine storm”.

Most individuals infected with SARS-CoV-2 are either asymptomatic or suffer mild symptoms. Severe cases may entail the risk of death due to acute respiratory distress syndrome (ARDS), cardiovascular damage, multiple organ failure or a hypercoagulable state, involving both microangiopathy and systemic coagulation defects [6,7]. These lethal outcomes result from direct effects of the virus on physiology [6,7], as well as excessive immune activation, also known as a “cytokine storm” [8]. It is well established that cytokine storms constitute a major cause of morbidity in viral-related diseases, such as SARS-CoV and the Middle East respiratory syndrome (MERS)-CoV, and likely also in COVID-19 [8,9]. High levels of IL-6, a hallmark cytokine of cytokine storms, at hospital admission were found to be associated with high COVID-19 mortality [6,7,8]. The interpersonal differences that bring about a deadly cytokine storm are not yet entirely known [10].

Based on current understanding, SARS-CoV-2 viruses enter the respiratory tract and infect epithelial cells and innate immune cells (specifically, lung macrophages and dendritic cells) [7]. An effective response is produced when, upon sensing the virus, innate immune cells rapidly recruit adaptive immune cells, such as T and B cells, by releasing type-I interferons. Virus-specific T- and B-cells identify their cognate viral antigens, become activated and increase in numbers. The T cells then mobilize to kill infected cells, while the B cells secrete antibodies, which inactivate cell-free viruses, preventing viral spread to uninfected cells. In most cases, these rapid, pathogen-specific, adaptive-immunity-oriented responses effectively reduce viral loads until viral clearance, usually with low collateral damage [7,10]. 

In contrast, among patients undergoing a cytokine storm, the pathogen-specific choreography of the adaptive immune system is replaced by excessive mobilization of innate immunity cells [11,12]. The innate-immunity dominated response less-effectively destroys the virus meanwhile indiscriminately inflicting substantial collateral damage on bystander tissue. Delayed secretion of type-I interferons, which are critical for inhibiting viral replication and igniting adaptive immune responses, results in high viral load within the lung epithelium [12]. Innate immunity cells, which sense the high viral load, secrete chemokines, such as CCL-2, CCL-3, and CCL-5, which attract additional innate immune cells to the lungs. In turn, the lung epithelium and the newly infiltrating innate immunity cells secrete large amounts of chemokines and cytokines, such as interleukin (IL)-1β, IL-6, IL-8, IL-10, interferon gamma (IFN-γ), and tumor necrosis factor-α (TNFα), attracting and activating more innate immunity cells. This positive feedback loop initiates a cytokine storm, which can lead to acute lung injury, ARDS, multiple-organ failure, and death [6,11,12,13]. Once a cytokine storm ignites, adaptive cells, too, may take part in the failed responses. T helper cells responding in an exaggerated Th1-type response may recruit macrophages and dendritic cells and activate them. Cytotoxic T lymphocytes (CTL) inefficiently killing infected cells lead to prolonged activation of T cells, which could trigger a cascade of inflammatory tissue damage [13]. Taken together, a delayed, pathogen-non-specific, innate-immunity-dominated response to a virus may kindle and amplify a life-threatening cytokine storm.

**Figure 1:**
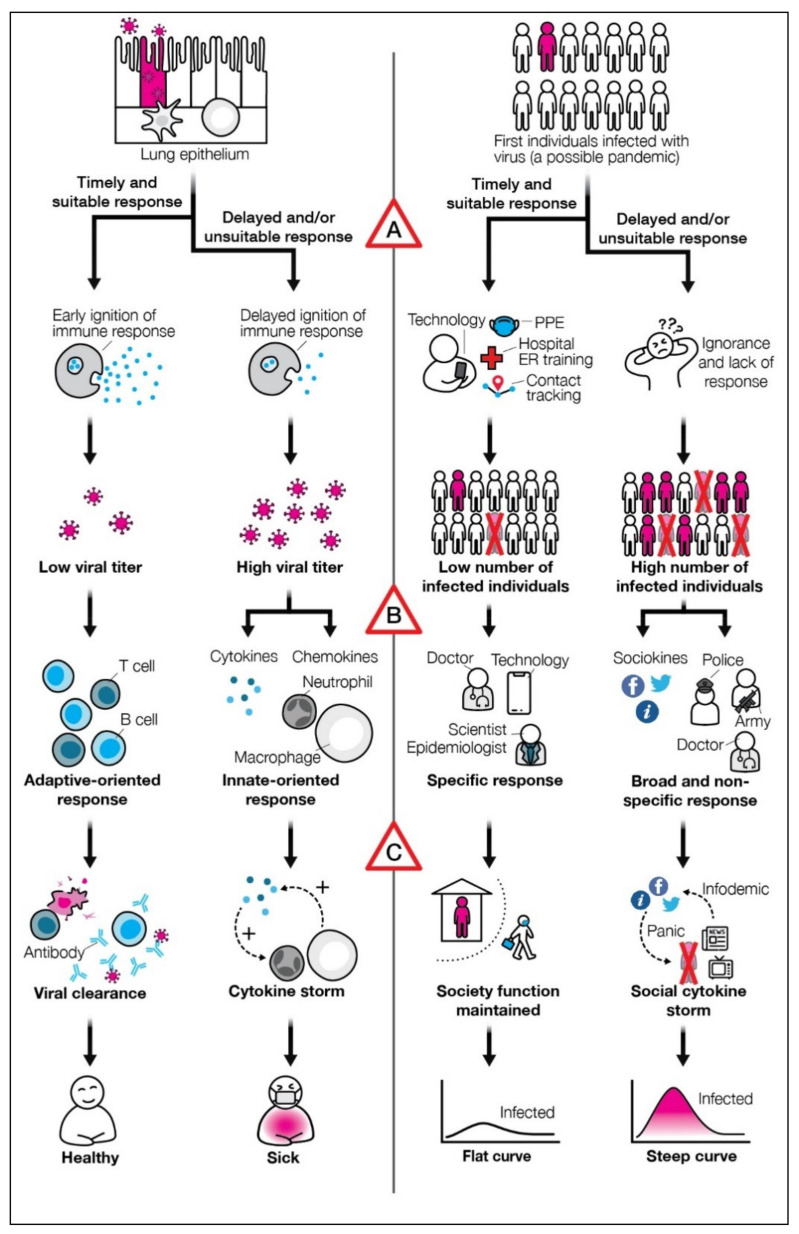
Parallels between individual and societal effective and ineffective responses to an invading pathogen. Triangles denote the factors determining the effectiveness of societal response to a pathogen. (**A**)—The quick employment of adaptive-like strategies to cope with a disease spreading throughout the society. (**B**)—Enabling productive coaction within the society or globally, by employing effective decision-making processes. (**C**)—Quick inhibition of positive feedback loops generated by hazardous or false information.

Surprising parallels are found between a harmful immunological cytokine storm, generated in response to a pathogen, and a social cytokine storm observed in the socio-political response to the same illness (see Figure 1). 

A societal failure to quickly initiate a response suited to the unique threat (similar to the delayed ignition of pathogen-specific adaptive responses via type-I IFNs) leads to a rapid increase in infected individuals and casualties (similar to an increased viral load). This sharp increase in sick individuals may overwhelm health systems. 

Similar to the positive feedback loop formed by the self-feeding cellular communication molecules (cytokines), the “social cytokines” (termed here “sociokines”), which arise from excessive media infodemic, misinformation, and fake news [14] are amplified throughout society. The surfeit of accurate and inaccurate information drives personal and societal uncertainty, panic, and destructive behavior [14].

In response to the public distress, governments are driven to impose full lockdowns enforced by the police or the military (similar to pathogen-non-specific responses), thus compromising the socioeconomic health of the society. Broad, pathogen-non-specific responses such as protracted and repeated full lockdowns compromise many critical societal functions, including business, travel, culture, and various civil liberties [15,16]. 

Taken together, a delayed, pathogen-non-specific response to a pandemic in a society may lead to excessive direct damage (sickness and death), local failure of healthcare systems (similar to ARDS) and subsequently to widespread socioeconomic consequences (similar to multiple-organ failure).

The recognition of parallels between an individual and a society dates back to the founders of modern sociology Comte and Spencer [17]. Hallpike and others have paralleled various societal institutions to organs—a government was represented by the brain whereas individuals were represented by cells [18]. The biological analogy of the society was used also by the Contagion theory of collective behavior. The theory suggests a “viral-like” transmission of irrational ideas, attitudes, emotions, and behaviors between people when engaged in a group, supporting the onset of social turmoil. Evidence has supported some facets of the Contagion theory. For instance, obesity, a complex physiological and social phenomenon, was shown to be propagated through social contagion-like processes [19]. These processes include contagious emotions (e.g., depression, sense of security) and unhealthy behaviors (e.g., the lack of physical activity) which spread within close social ties and in social networks [20].

The parallels between a sick individual and a society harboring many sick individuals underlie the fractal-like relationship between these two self-similar health-preserving complex systems, in which a society echoes the traits of its lower level of complexity (the individual). The understanding that similar dynamics govern personal or societal responses to a pathogen can serve as a missing link that puts into a broader context various societal responses to a pathogen, and may help in identifying critical factors among the societal response to a pathogen that govern the effectiveness of the responses (Figure 1, triangles marked A–C). These include: A—How quickly are adaptive-like strategies employed to cope with the pathogenic threat; B—Is a productive co-action or interaction within the society enabled through employing effective decision-making processes; C—How quickly are hazardous or false, self-feeding information loops inhibited. 

## 2. Factors Determining the Effectiveness of Societal Response to a Pathogen 

### 2.1. Quick Employment of Adaptive Strategies to Deal with the Pathogenic Threat

The only strategies capable of resolving a pandemic with minimal societal damage are ones resembling adaptive, pathogen-specific responses (Figure 1A). Adaptive immune responses generate immune memory, which enables a quicker, more effective response upon re-exposure to the pathogen (inevitable during long-lasting pandemics). Societal (and global) adaptive pathogen-specific responses are the development of vaccines or therapeutics. Such pre- or post-exposure solutions, respectively, have solved health threats more menacing than COVID-19, most notably HIV, smallpox, and polio. Yet, until the development of safe and effective vaccines [21] or therapeutics, and their effective worldwide distribution, viral spread among all societies is inevitable.

Innovative technologies, however, may provide temporary, adaptive-like solutions to combat a pathogen before vaccines or therapeutics are developed. Such technologies include analyses of local mobility and immigration by means of mobile phones, wearables or big-data-related technologies, which enable contact tracing and early identification of infected clusters [22]. Innovative technologies, like their immunological counterparts, can only be effective if systemically applied to all parts of the society, leaving no safe-havens where the virus can dwell and propagate.

Different societies have different capabilities to adaptively-cope with a pandemic. “Immune competent” societies have the resources and capabilities to withstand a pandemic. Effective, adaptive-like societal responses are comprised of: (1) Quick ignition of pathogen-specific healthcare-preparedness and enforcement of pathogen-suited social response guidelines (similar to early secretion of type-I interferons); (2) Rapid identification and restriction of local outbreaks by means of mass testing (similar to the production of virus-specific antibodies); (3) Rapid containment of the disease by contact tracing and quarantining exposed individuals or communities (similar to activation of anti-viral T-cells which neutralize infected cells); and (4) Learning how to deploy quicker, more effective responses upon virus resurgence (similar to the development of an immune memory). 

For instance, South Korea quickly adapted the health responses that it had formulated following the MERS-CoV epidemic (similar to memory response). The South Korean government met the outbreak with considerable capacity to perform mass testing and contact tracing, and it enforced social distancing guidelines without implementing full lockdown. These measures resulted in a successful containment of the number of casualties, after several waves of infection, to approximately 2000 (by May 2021), averting excessive socioeconomic damage [23,24].

Even in immune competent countries, however, “immune ignorance” (not recognizing the pathogen or its possible impact on society) may result in a delayed and inadequate response. For example, the U.S. saw its first confirmed COVID-19 case one day after it was detected in South Korea, yet it had tragically delayed full mobilization of its healthcare system [25]. This led to high casualty rates, especially in New York City (NYC). After casualties had mounted, the NYC healthcare systems became overwhelmed and could not adequately accommodate all of those needing treatment. By that time, public fear had mounted and a full nationwide lockdown was implemented: major health damage had been wreaked, driving major socioeconomic damage that may have long-term effects [26]. Hence, potentially effective measures, if taken in delay, could drive health, social, and economic systems to spiral out of control.

An “immune-compromised” society, like an immune-compromised individual, lacks the necessary resources or capabilities to adaptively deal with infection. Ecuador’s under-resourced healthcare system is an example of this, with the resulting high death tolls and a collapse of burial systems [27]. Without international aid, such societies will have to resort to pathogen non-specific innate-like responses, such as full lockdowns. 

While short-term full lockdowns are necessary and effective to reduce deaths from COVID-19, and to prevent an overwhelmed healthcare system, repeated and long lockdowns drive huge loss of income, increase in unemployment, increase in mental illness, decrease in confidence in government, and disrupt education [15]. No society can remain hermetically or indefinitely locked-down without sustaining unendurable socioeconomic damage.

Notably, before the onset of modern medicine or the discovery of vaccines, pandemics, such as the Black Death or smallpox, recurred over many generations. Taken together, without the development of adaptive-like, pathogen-specific, memory-enabling responses, societies would inevitably experience recurrent waves of disease. 

### 2.2. Enabling Productive Coaction within the Society and between Societies by Employing Effective Decision-Making Processes 

Productive interaction and coaction of the different arms of the immune system are vital for effective anti-pathogen immune responses (Figure 1B). System-level immune “decisions” regarding if and how to respond to a pathogen depend on effective collection of information on the pathogen, as well as structured decision-making processes taking place usually between innate and adaptive immune components. Dendritic cells (DC, innate immunity cells) identify invading pathogens using receptors to broad pathogen-associated molecular patterns (PAMPs). DC then present pathogen-derived antigens, with appropriate costimulatory signals, to adaptive immunity cells such as helper T cells which, in turn, license the DC to activate additional pathogen-specific immune effectors as cytotoxic T cells (CTL) or B cells. Such hardwired, structured decision-making processes can ignite effective anti-pathogen responses. These effective anti-pathogen capabilities, are preserved once the initial infection had subsided, enabling quicker, more effective memory responses upon pathogen resurgence.

Similarly, productive collaboration and coaction between different information-gathering, decision-making, and effector arms of the society are essential for an effective societal response to a pathogen [28]. Such a response requires effective collection, distribution and analysis of information about the pathogen by scientists and physicians. Gathered information should then be processed by healthcare policymakers and politicians to generate a suitable response to the specific pathogenic threat. The defined healthcare policy should enable healthcare systems, law enforcement agencies, and social welfare systems to effectively work together. Upon completing an initial effective response to the pathogen, the interacting arms should consolidate their gained knowledge to enable quicker and more efficient responses upon pathogen resurgence. 

Global pandemics illustrate that effective collaborations are also required in levels higher than those of a single society. Since no society can remain permanently confined, quick and adequate flow of accurate information between societies, together with global decision-making policies could significantly reduce initial and on-going viral spread between countries. Such higher-level coaction could also assist global efforts to quickly develop vaccines, therapeutics and technological solutions that could flatten the global pandemic curve [22,28]. 

### 2.3. Quick Inhibition of Positive Feedback Loops Generated by Hazardous or False Information

Immune cytokine storms occur when a hazardous positive feedback loop is formed. The innate cells secrete cytokines and chemokines, which summon and then activate additional innate cells [8]. Therapeutics, such as immunosuppressive dexamethasone, administered to the appropriate patients at the appropriate timing may mitigate or curb an ongoing cytokine storm [29].

A social cytokine storm may occur if pathogen non-specific innate-like responses are deployed (Figure 1C). In a society, it is important to install mechanisms to convey reliable information to the public and to quickly identify and contradict incorrect and destructive information, which, in times of uncertainty, may generate hazardous self-feeding information loops. Massive amount of misinformation has surfaced with regard to the means of transmission of COVID-19 (flies, mosquitos, 5G networks), its prevention (hot/cold baths, garlic, hydroxychloroquine), and its treatment (methanol, bleach, consumption of strong spirts) [30]. Similar hazardous self-feeding information loops are endangering the acceptance and widespread use of the approved COVID-19 vaccines.

The responsibility to quickly and effectively respond to misinformation and predatory quackery lies with the scientific community, law enforcement agencies, and with policymakers who can ally to install a trusted communication channel with the public [31]. Apt societal responses to misinformation should consist of two main actions. First, to provide calm, up to date, scientifically-based messaging by public health authorities, to contradict quackery and dissipate false claims of prevention, treatment, and cure. Second, to take robust and well-publicized legal action against those responsible for the promotion of quackery and misinformation [32]. Sadly, much of the misinformation during the COVID-19 pandemic was conveyed by persons of influence, including politicians, community leaders, and religious leaders. Institutional legitimacy and public trust had plummeted in numerous societies including the U.S., Brazil, Israel, and Belarus [33,34,35]. In contrast, societies that have responded quickly and effectively, maintaining legitimacy and public trust, including Singapore, New Zealand, and South Korea, were able to sustain public adherence to health policies and to ease economic and social upheaval.

## 3. Conclusions

The adaptive immune system was sculpted in a continuous battle to protect individual animals, and species over the last 500 million years. Learning from field-tested effective anti-pathogen immune responses, policymakers and scientists could adopt similar adaptive-like approaches, in order to reduce the direct damage associated with emerging pandemics such as COVID-19 and to avert avoidable social cytokine storms and their ensuing socioeconomic impairment.

## Data Availability

Not applicable.
